# A modified partition of peripapillary atrophy in high myopia based on optical coherence tomography

**DOI:** 10.3389/fmed.2026.1759553

**Published:** 2026-02-18

**Authors:** Kaiyao Chi, Biao Li, Tong Guo, Daoxi Lei, Yanlin Zheng, Huili Li

**Affiliations:** 1 The First Affiliated Hospital of Chongqing University of Chinese Medicine, Chongqing, China; 2 Hospital of Chengdu University of Traditional Chinese Medicine, Chengdu, China; 3 Chengdu University of Traditional Chinese Medicine, Chengdu, China; 4 Chongqing University of Chinese Medicine, Chongqing, China; 5 Sichuan Provincial People's Hospital, Chengdu, China

**Keywords:** high myopia, myopia, optical coherence tomography, pathological myopia, peripapillary atrophy zones, disease progression, disease prognosis

## Abstract

**Objective:**

This study investigates morphological changes in peripapillary atrophy (PPA) in high myopia (HM) patients, aiming to refine PPA zoning characteristics to enhance clinical diagnosis and treatment of HM.

**Design:**

This study is categorized as a retrospective cross-sectional model.

**Methods:**

We retrospectively reviewed 172 patients (316 eyes) with comprehensive medical records, categorizing them into a pathological myopia (PM) cohort—subdivided into PM1 (≥30 mm), PM2 (≥28 and <30 mm), PM3 (<28 mm)—and a simple HM group, based on fundus pathology and axial length (AL). We collected data on spherical equivalent (SE), best corrected visual acuity (BCVA), intraocular pressure (IOP), AL, and posterior optical coherence tomography (OCT). For posterior OCT, we manually measured improved landmark features based on the original PPA zoning: Scleral Curvature Angle (SCA), Scleral Flange Curvature Angle (SFCA), PPA1-4 zone, scleral flange length (SFL), and mean scleral flange thickness (MSFT). The Bland–Altman method assessed measurement consistency, and statistical comparisons were made of the modified PPA feature parameters across the groups.

**Results:**

An increase in AL and progression from simple HM to PM were associated with increases in age, AL, and IOP, along with decreases in BCVA and SE across all groups. The parameters SCA, SFCA, and PPA1-4 zones showed a general increase, whereas MSFT differences were not statistically significant (*p* > 0.05). In multiple factor linear regression analysis, PPA1 and SCA were strongly correlated with BCVA, AL, and SE (*p* < 0.01).

**Conclusion:**

The modified PPA partition based on OCT imaging identifies 6 key zoning features: PPA1-4, SCA, and SFCA. These are correlated with longer AL and advanced fundus changes in HM patients. In particular, PPA1 and SCA emerge as vital indicators for assessing HM conditions using OCT imaging.

## 1 Introduction

Myopia, affecting nearly 2 billion individuals globally (28.3%), including 277 million (4.0%) with high myopia (HM), is among the world’s most prevalent ocular conditions. Projections indicate an increase to 4.76 billion myopic individuals and 1 billion with HM by 2050 (1). Pathologic myopia (PM), an advanced stage of HM, is characterized by excessive elongation of the eyeball’s sagittal axis. This elongation results in mechanical stretching and thinning of the posterior eye wall, thereby affecting the retina, choroid, and sclera, leading to degenerative alterations and potential vision loss.

Optical coherence tomography (OCT) is a pivotal tool for visualizing fundus characteristics in HM patients. Notably, the posterior scleral wall in HM deviates from the flat appearance seen in healthy eyes, exhibiting an incomplete oval shape. Peripapillary atrophy (PPA), another hallmark in HM, is classically segmented into four zones (*α*, *β*, *γ*, and *δ*), with the latter two zones significantly associated with HM (2). However, clinical observations reveal difficulties in discerning the α and δ zones in OCT images, and the β and γ zones often appear as discontinuous lines, diminishing the diagnostic utility of PPA features for assessing HM.

Our review of OCT data from HM diagnoses over the past year identified two notable anatomical landmarks associated with PPA: a sclera bending near the Bruch’s membrane (BM) termination observed in most simple HM and nearly all PM cases, and a curvature near the dura mater-scleral flange boundary present in a minority of simple HM and the majority of PM patients. Collaborations with ophthalmologists suggest that these OCT-identified landmarks may enhance preliminary HM assessments. This study aims to investigate the relevance of these anatomical features to HM progression, offering insights that could refine early diagnostic approaches.

## 2 Methods

### 2.1 Participants

This retrospective study reviewed 172 patients (316 eyes) diagnosed with HM and documented in the electronic medical records at the Ophthalmology Outpatient Department of the First Affiliated Hospital of Chongqing University of Chinese Medicine from April to December 2023. All medical information was verified for completeness and reliability. The research was approved by the Ethics Committee of the First Affiliated Hospital of Chongqing University of Chinese Medicine (No. 2025-IIT-HY-19) in accordance with the principles of the Declaration of Helsinki and all applicable laws in our country.

### 2.2 Diagnostic criteria

Given the absence of universally accepted criteria for HM, this study adopted the evidence-based consensus recommendation (3): HM is defined as a spherical equivalent (SE) o ≤ −6.00 diopters or an axial length (AL) of ≥ 26.5 mm.

PM classification incorporates fundus changes indicative of pathological progression in HM patients, including myopic atrophic maculopathy (MAM), myopic traction maculopathy (MTM), myopic neovascular maculopathy (MNM), or posterior staphyloma presence (4), with grading according to the latest Atrophy–Neovascularization–Traction (ANT) classification system for defining MAM (grade A2 and above), MTM (grade T1 and above), and MNM (grade N1 and above) (5), as shown in Table 1.

**Table 1:** ANT classification of PM.

Classification (level)	Atrophy, A	Neovascularization, N	Traction, T
0	No myopic retinal lesions	No myopic macular neovascularization (MNV)	No macular schisis
1	Tessellated fundus	Lacquer cracks	Inner or outer foveoschisis or lamellar macular hole
2	Diffuse chorioretinal atrophy	a: Active choroidal neovascularization (CNV) s: Scar or Fuch spot	Inner and outer foveoschisis
3	Extrafoveal patchy chorioretinal atrophy	–	Foveal detachment
4	Foveal patchy chorioretinal atrophy	–	Full-thickness macular hole
5	–	–	Macular hole + retinal detachment

### 2.3 Inclusion criteria

Inclusions were made for individuals who were required to: (1) be 18 years or older; (2) meet the above HM (including PM) diagnostic criteria; (3) have a complete set of observation data; (4) provide consent after being contacted via phone, along with their family’s consent.

### 2.4 Exclusion criteria

Exclusions were made for individuals with: (1) a history of eye trauma, refractive, or intraocular surgery; (2) other retinal diseases identified in addition to PM upon examination; (3) conditions or systemic diseases potentially affecting fundus imaging, such as keratitis, glaucoma, cataracts, or severe organ-specific or systemic illnesses; (4) medication use known to influence fundus appearance, like hydroxychloroquine; (5) vitreous cavity injections of corticosteroids or anti-vascular endothelial growth factor drugs within 3 months before examination.

### 2.5 Grouping

According to data from 9,161 visits to 1,877 HM patients reported by Du et al., the average AL was 29.66 ± 2.20 mm, and a baseline AL ≥ 28.15 mm was an extremely strong risk factor (6). Therefore, in this study, patients were classified into a PM group, subdivided into PM1 (AL ≥ 30 mm), PM2 (AL ≥ 28 mm and < 30 mm), PM3 (AL < 28 mm), and a simple HM group based on PM-related fundus changes and AL.

### 2.6 Observation indicators

A comprehensive ophthalmological evaluation was conducted for each patient, including measurements of spherical equivalent (SE) (D), best corrected visual acuity (BCVA) using the Early Treatment Diabetic Retinopathy Study (ETDRS) Logarithm of the Minimum Angle of Resolution (logMAR) chart (count), AL with IOL Master 500 (mm), intraocular pressure (IOP) with Topcon CT-60 (mm Hg), and OCT using Heidelberg Eye Explorer.

### 2.7 OCT image acquisition

**Figure 1:**
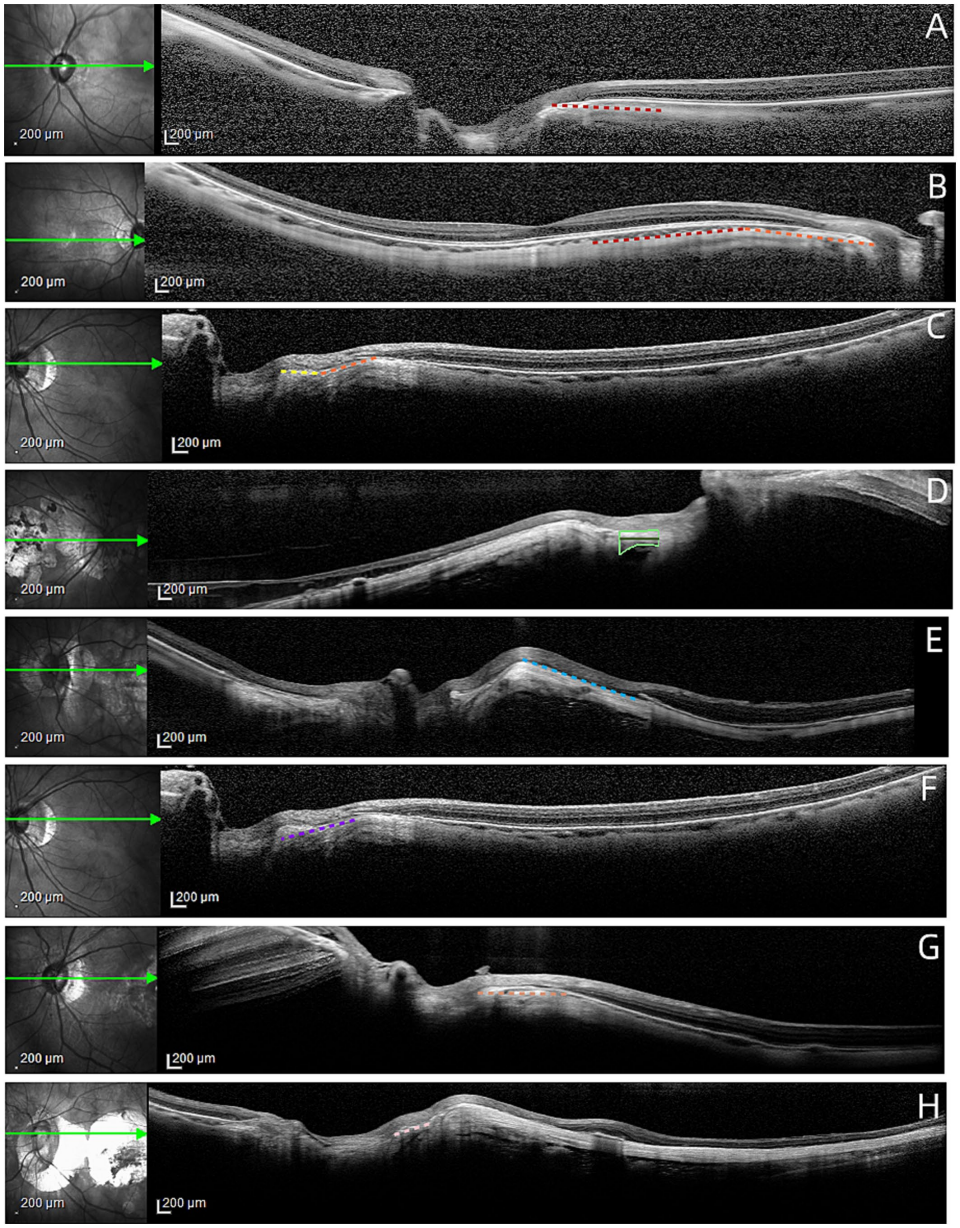
Definition of the OCT-based PPA modified zonation-related parameters. **(A)** HM patient without SCA and SFCA (red dashed line: normal peripapillary sclera radian); **(B)** HM patient with SCA only (red dashed line: normal peripapillary sclera radian; orange dashed line: peripapillary sclera curvature; the deflection of the orange dashed line relative to the red dashed line is SCA); **(C)** HM patients with both SCA and SFCA (orange dashed line: peripapillary scleral curvature; yellow dashed line: scleral flanges curvature; the deflection of the yellow dashed line relative to the orange dashed line is SFCA); **(D)** Measurement of MSFT (light green line: SFA; dark green line: SFL); **(E)** Definition of PPA1 (blue dashed line: when BM is interrupted at the temporal side of SCA, the line from BM interruption to SCA vertex); **(F)** Definition of PPA2 (purple dashed line: the line from BM interruption to optic disc edge when BM was interrupted nasal to SCA); **(G)** Definition of PPA3 (brown dotted line: line from SCA vertex to optic disc edge); **(H)** Definition of PPA4 (pink dotted line: line from apex of SFCA to optic disc edge).

For all patients, the frontal and mandibular medians were aligned at the midpoint of the device to ensure the standardization of the scanning direction. OCT images were obtained using the data acquisition protocols of Topcon three-dimensional (3D)-OCT and Deep Range Imaging (DRI)-OCT Atlantis in Enhanced Depth Imaging (EDI) mode, focusing on high-quality horizontal scans through the optic disc center. For image analysis, we selected the “Small HRA image, large OCT image” mode, adjusted the image display from the default “1:1 pixel” to “1:1 μm,” and magnified images to 600% for detailed measurement. The “Equally large HRA and OCT images” mode was used for analyzing infrared fundus images. Measurements were conducted independently by two investigators (Tong Guo and Daoxi Lei), focusing on the following structures, as illustrated in Figure 1.

Scleral Curvature Angle (SCA): Positioned between the fovea of the macula and the optic disc, the SCA delineates the crucial juncture where the normal curvature of the sclera (indicated by the red dashed line) transitions into abnormal curvature (indicated by the orange dashed line), bending away from the eyeball toward the orbit. Utilizing the Angle tool mode of ImageJ, we measured the angle between the horizontal line of the scleral inner boundary on the nasal abnormal curvature and the horizontal line of the scleral inner boundary on the temporal normal curvature at this critical juncture. In cases where there was a secondary bending of the sclera on the nasal side of the SCA, the scleral horizontal line closest to the bending angle was selected. A normal SCA is approximately 0 degrees.

Scleral Flange Curvature Angle (SFCA): Positioned between the SCA and the optic disc, the SFCA denotes the critical juncture where the scleral flange (highlighted by the yellow dashed line) deviates laterally from its abnormal orientation relative to the orange dashed line. This deviation is recorded as a positive angle when the scleral flange turns toward the eyeball and as a negative angle when it turns toward the orbit. Employing the Angle tool mode of ImageJ, we measured the angle of deviation of the horizontal line of the nasal scleral flanges from the horizontal line of the temporal abnormal curvature. Typically registers around 0 degrees in individuals with normal ocular anatomy.

PPA1–4 zones: Defined based on the absence of BM and their relative positions to SCA and SFCA. Measurements were made using the software’s built-in ruler tool: PPA1: The distance from BM’s end to SCA’s apex, on the temporal side of SCA. PPA2: The distance from BM’s end to the optic disc edge, on the nasal side of SCA. PPA3: The distance from SCA’s apex to the optic disc boundary. PPA4: The distance from SFCA’s apex to the optic disc edge.

Scleral Flange Length (SFL): Identified by the clear boundary of the scleral flange, measuring from nasal to temporal borders using the software’s built-in ruler tool.

Mean scleral flange thickness (MSFT): For eyes with clear PPA4 and SFL visibility, the scleral flange area (SFA) directly beneath these landmarks was measured employing the software’s built-in “selection measurement tool. MSFT was calculated by dividing the SFA by PPA4 or SFL.

### 2.8 Statistical analysis

Data analysis was conducted using the Scientific Platform Serving for Statistics Professional (SPSSPRO). For categorical data, the χ^2^ test was employed. The Shapiro–Wilk test assessed the normality of continuous variables, while the Hartley test evaluated variance homogeneity. When data were normally distributed and homoscedastic, one-way analysis of variance (ANOVA) was used, followed by *post hoc* multiple comparisons using the LSD method. For data not meeting these criteria, the Kruskal–Wallis H test was applied, with the Mann–Whitney U test for subsequent *post hoc* multiple comparisons. Results were interpreted as two-sided, with *p*-values less than 0.05 considered statistically significant and those less than 0.01 considered highly significant.

## 3 Results

### 3.1 General information

The results reveal pronounced differences across the four groups in age, BCVA, AL, SE, IOP (*p* < 0.01), and gender (*p* < 0.05), as shown in Table 2.

**Table 2:** General information.

Statistical object	PM1	PM2	PM3	HM	*p*
Number	74	83	79	80	-
Age (years, x̄±SD)	60.959 ± 13.334	56.723 ± 13.257	55.57 ± 13.531	51.075 ± 14.628	0.000**
Sex (female/male)	30/44	34/49	20/59	38/42	0.031*
BCVA (count, MD ± SD)	53 ± 27.865	74 ± 23.382	77 ± 16.854	86 ± 4.278	0.000**
AL (mm, MD ± SD)	31.485 ± 1.644	28.96 ± 0.571	27.51 ± 0.518	27.04 ± 0.839	0.000**
SE (D, MD ± SD)	−15.5 ± 4.155	−11.5 ± 1.877	−8.5 ± 1.789	−8 ± 2.085	0.000**
IOP (mm Hg, MD ± SD)	18 ± 2.745	17 ± 2.789	16 ± 2.361	16 ± 2.262	0.000**

*p < 0.05; **p < 0.01. MD, mean deviation; SD, standard deviation.

The mean age order was PM1 > PM2 > PM3 > HM. *Post hoc* multiple comparisons were used to analyze age, among which HM and (PM1 and PM2) showed extremely significant differences (*p* < 0.01), PM3 and (PM1 and HM) showed significant differences (*p* < 0.05), as shown in Table 3.

**Table 3:** *Post hoc* multiple comparisons of age.

*p*	PM1	PM2	PM3	HM
PM1	–	0.054	0.016*	0.000**
PM2	0.054	–	0.593	0.009**
PM3	0.016*	0.593	–	0.039*
HM	0.000**	0.009**	0.039*	–

*p<0.05, **p<0.01.

*Post hoc* multiple comparisons of BCVA, AL, SE, and IOP found that the median order of BCVA was: HM > PM3 > PM2 > PM1, and there were extremely significant differences between PM1 and (PM2, PM3, and HM), HM and (PM2 and, PM3) (*p* < 0.01), and PM2 and PM3 (*p* < 0.05). The median of AL was PM1 > PM2 > PM3 > HM, and there were significant differences between PM1 and (PM2, PM3, and HM), PM2 and (PM3, HM) (*p* < 0.01). The median of SE was HM > PM3 > PM2 > PM1, and there were significant differences between PM1 and (PM2, PM3, and HM), PM2 and (PM3 and HM) (*p* < 0.01). The median order of IOP was PM1 > PM2 > PM3 = HM, and there was a very significant difference between (PM1 and PM2) and (PM3 and HM) (*p* < 0.01), as shown in Table 4.

**Table 4:** *Post hoc* multiple comparisons of BCVA, AL, SE, and IOP.

Two independent samples		BCVA		AL		SE		IOP	
Group 1	Group 2	Median difference	*p*	Median difference	*p*	Median difference	*p*	Median difference	*p*
PM1	PM2	21	0.000**	2.525	0.000**	4	0.000**	1	0.390
PM1	PM3	24	0.000**	3.975	0.000**	7	0.000**	2	0.000**
PM1	HM	33	0.000**	4.445	0.000**	7.5	0.000**	2	0.000**
PM2	PM3	3	0.041*	1.45	0.000**	3	0.000**	1	0.001**
PM2	HM	12	0.000**	1.92	0.000**	3.5	0.000**	1	0.000**
PM3	HM	9	0.000**	0.47	0.404	0.5	1.382	0	1.847

*p<0.05, **p<0.01.

### 3.2 PPA related parameters

**Figure 2:**
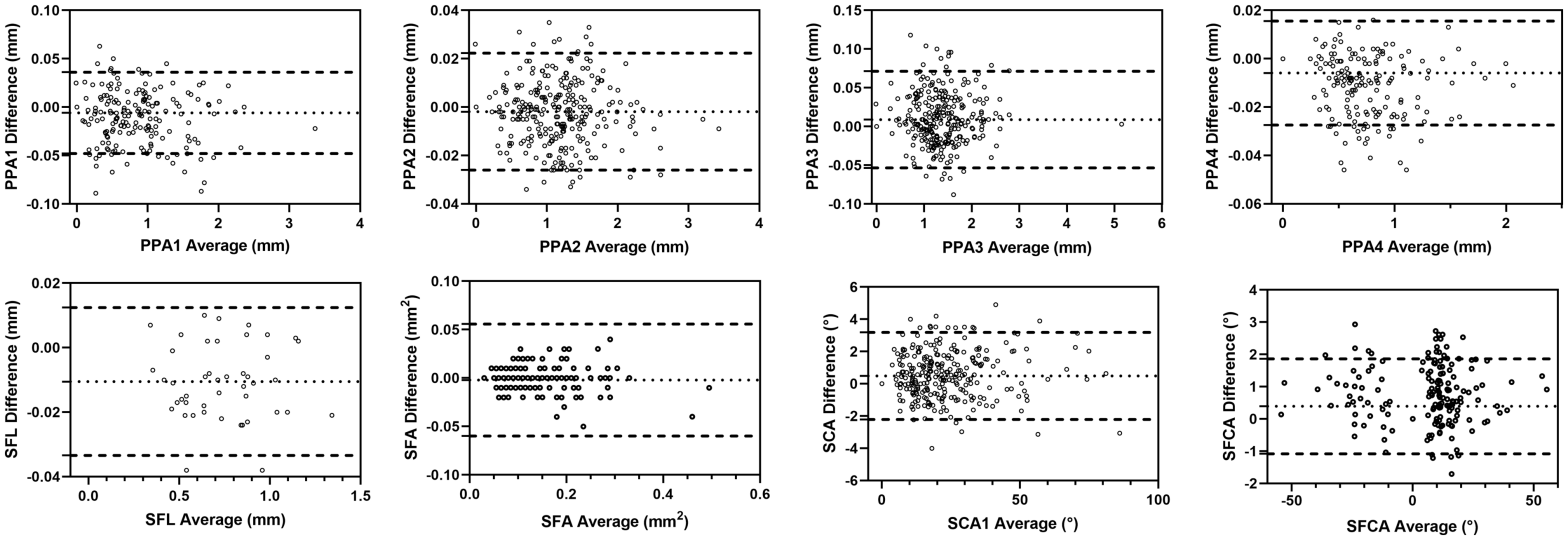
Bland–Altman plot of each hand-measured feature of PPA.

Bland–Altman plot analysis for interobserver agreement indicated no systematic bias between measurements, ensuring reliability in the data collection process, as shown in Figure 2.

Four groups in the PPA1, PPA2, PPA3, PPA4, SCA, SFCA, and |SFCA| exist significant differences (*p* < 0.01), are shown in Table 5.

**Table 5:** PPA related parameters (x̄±SD).

Statistical object	PM1	PM2	PM3	HM	*p*
Number	74	83	79	80	
PPA1	0.635 ± 0.729	0.427 ± 0.533	0 ± 0.544	0 ± 0.405	0.000**
PPA2	1.332 ± 0.408	1.225 ± 0.61	0.899 ± 0.625	0.353 ± 0.479	0.000**
PPA3	1.383 ± 0.427	1.404 ± 0.69	1.212 ± 0.6	0.744 ± 0.624	0.000**
PPA4	0.511 ± 0.391	0.57 ± 0.511	0 ± 0.446	0 ± 0.352	0.000**
SCA	28 ± 17.521	21 ± 14.641	14 ± 10.47	9 ± 9.758	0.000**
SFCA	7.5 ± 17.292	7 ± 14.399	0 ± 10.456	0 ± 10.266	0.001**
|SFCA|	11.5 ± 12.795	11 ± 10.179	0 ± 8.609	0 ± 9.256	0.000**

*p<0.05, **p<0.01.

Multiple comparisons after ANOVA of PPA1, PPA2, PPA3, PPA4, SCA, SFCA, and |SFCA| found that the median order of PPA1 was: PM1 > PM2 > PM3 = HM, and there were significant differences between PM1 and (PM3, HM), PM2 and HM (*p* < 0.01), and PM1 and PM2 (*p* < 0.05). The median order of PPA2 was PM1 > PM2 > PM3 > HM, and there were significant differences between HM and (PM1, PM2, and PM3) and PM3 and (PM1 and PM2) (*p* < 0.01). The median order of PPA3 was PM2 > PM1 > PM3 > HM, in which there was a significant difference between HM and (PM1, PM2, and PM3) (*p* < 0.01), and PM3 and (PM1 and PM2) (*p* < 0.05). The median order of PPA4 was: PM2 > PM1 > PM3 = HM, in which there were significant differences between HM and (PM1 and PM2), PM2 and PM3 (*p* < 0.01), and PM3 and HM (*p* < 0.05). The median order of SCA was PM1 > PM2 > PM3 > HM, and there were significant differences among all groups (*p* < 0.01). The median order of SFCA was: PM2 > PM1 > PM3 = HM, there was a significant difference between HM and (PM1 and PM2) (*p* < 0.01), and there was a significant difference between PM3 and PM1 (*p* < 0.05). |SFCA| has a median ranking as follows: PM2 > PM1 > PM3 = HM, and there is a very significant difference between (PM1 and PM2) and (PM3 and HM) (*p* < 0.01), as shown in Table 6.

**Table 6:** *Post hoc* multiple comparisons of PPA-related parameters.

Two independent samples		PPA1		PPA2		PPA3		PPA4		SCA		SFCA		|SFCA|	
Group 1	Group 2	Median difference	*p*	Median difference	*p*	Median difference	*p*	Median difference	*p*	Median difference	*p*	Median difference	*p*	Median difference	*p*
PM1	PM2	0.208	0.019*	0.107	0.383	0.021	1.506	0.059	0.784	7	0.004**	0.5	1.108	0.5	1.32
PM1	PM3	0.635	0.000**	0.433	0.000**	0.171	0.024*	0.511	0.058	14	0.000**	7.5	0.044*	11.5	0.002**
PM1	HM	0.635	0.000**	0.979	0.000**	0.64	0.000**	0.511	0.000**	19	0.000**	7.5	0.001**	11.5	0.000**
PM2	PM3	0.427	0.136	0.326	0.001**	0.192	0.017*	0.57	0.008**	7	0.000**	7	0.111	11	0.003**
PM2	HM	0.427	0.002**	0.872	0.000**	0.66	0.000**	0.57	0.000**	12	0.000**	7	0.003**	11	0.000**
PM3	HM	0	0.473	0.546	0.000**	0.468	0.000**	0	0.049*	5	0.001**	0	0.386	0	0.053

*p < 0.05, **p < 0.01.

### 3.3 MSFT

A total of 153 eyes with OCT images that clearly identified SFA and SFL or PPA4 were selected for analysis, MSFT = SFA/(SFL or PPA4). The results showed no significant difference in MSFT among the four groups (*p* > 0.05), as shown in Table 7.

**Table 7:** MSFT (x̄±SD).

Statistical object	PM1	PM2	PM3	HM	*p*
Number	38	46	39	30	
MSFT	0.195 ± 0.057	0.196 ± 0.07	0.205 ± 0.061	0.191 ± 0.052	0.785

*p<0.05, **p<0.01.

### 3.4 Correlation between PPA parameters and BCVA

Univariate linear regression showed that IOP, PPA1, PPA2, PPA3, and SCA had significant linear relationships with BCVA (*p* < 0.01), whereas SFCA had significant linear relationships with BCVA (*p* < 0.05). Subsequently, these 6 parameters were included in a multifactor linear regression, and the results showed that there was a very significant linear relationship between PPA1 and SCA, and between PPA1 and BCVA (*p* < 0.01), as shown in Table 8.

**Table 8:** Univariate and multifactorial linear regression models of the effects of PPA-related parameters on BCVA.

Variable	Univariate analysis				Multivariate analysis			
	B	*β*	*R* ^2^	*p*	B	β	R^2^	*p*
IOP	−1.671	−0.189	0.036	0.001**	0.241	0.027	0.17	0.69
PPA1	−10.607	−0.261	0.068	0.000**	−6.744	−0.167		0.005**
PPA2	−9.337	−0.249	0.062	0.000**	−0.734	−0.02		0.846
PPA3	−6.35	−0.179	0.032	0.001**	−2.364	−0.067		0.486
PPA4	−5.274	−0.099	0.01	0.08				
SCA	−0.575	−0.377	0.142	0.000**	−0.467	−0.297		0.000**
SFCA	−0.243	−0.136	0.018	0.016*	−0.031	−0.017		0.763

*p<0.05, **p<0.01.

### 3.5 Correlation between PPA parameters and AL

Univariate linear regression showed that IOP, PPA1, PPA2, PPA3, PPA4, and SCA had significant linear relationships with AL (*p* < 0.01), and SFCA had significant linear relationships with AL (*p* < 0.05). Subsequently, these seven parameters were included in multifactor linear regression, and the results showed that there was a very significant linear relationship between PPA1 and SCA and AL (*p* < 0.01), as shown in Table 9.

**Table 9:** Univariate and multifactorial linear regression models of the effects of PPA-related parameters on AL.

Variable	Univariate analysis				Multivariate analysis			
	*B*	*β*	*R* ^2^	*p*	*B*	*β*	*R* ^2^	*p*
IOP	0.217	0.281	0.079	0.000**	−0.009	−0.012	0.306	0.851
PPA1	1.079	0.303	0.092	0.000**	0.692	0.201		0.000**
PPA2	1.423	0.434	0.188	0.000**	0.566	0.178		0.058
PPA3	1.006	0.324	0.105	0.000**	0.216	0.072		0.42
PPA4	0.874	0.194	0.038	0.001**	−0.057	−0.013		0.832
SCA	0.067	0.5	0.25	0.000**	0.039	0.293		0.000**
SFCA	0.022	0.009	0.02	0.013*	0.006	0.037		0.504

*p<0.05, **p<0.01.

### 3.6 Correlation between PPA parameters and SE

Univariate linear regression showed that IOP, PPA1, PPA2, PPA3, and SCA had significant linear relationships with SE (*p* < 0.01), and PPA4 had significant linear relationships with SE (*p* < 0.05). Subsequently, these six parameters were included in multifactor linear regression, and the results showed that there was a very significant linear relationship between PPA1 and SCA and SE (*p* < 0.01), as shown in Table 10.

**Table 10:** Univariate and multifactorial linear regression models of the effects of PPA-related parameters on SE.

Variable	Univariate analysis				Multivariate analysis			
	*B*	*β*	*R* ^2^	*p*	*B*	*β*	*R* ^2^	*p*
IOP	−0.411	−0.265	0.07	0.000**	−0.054	−0.035	0.236	0.565
PPA1	−1.986	−0.279	0.078	0.000**	−1.45	−0.208		0.000^***^
PPA2	−2.543	−0.387	0.15	0.000**	−0.873	−0.135		0.167
PPA3	−1.89	−0.304	0.092	0.000**	−0.896	−0.147		0.116
PPA4	−1.252	−0.137	0.019	0.015*	0.576	0.063		0.29
SCA	−0.112	−0.419	0.176	0.000**	−0.054	−0.201		0.004^***^
SFCA	−0.024	−0.078	0.006	0.169				

*p<0.05, **p<0.01.

## 4 Discussion

### 4.1 PPA classic partition

**Figure 3:**
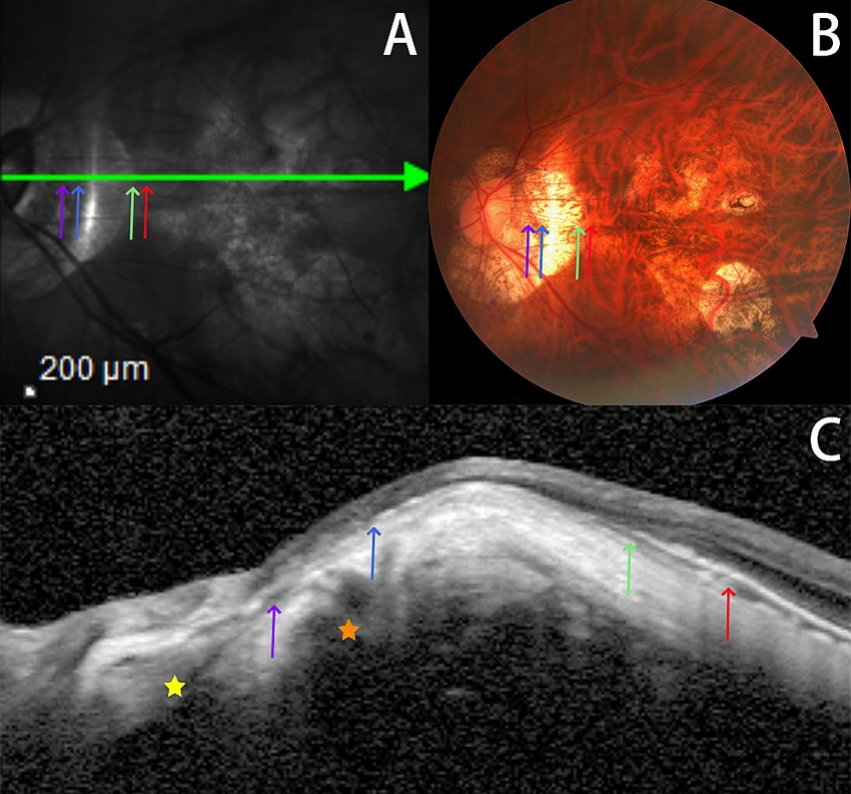
PPA classical partition. **(A)** Infrared fundus map; **(B)** Fundus color photos of the same patient; **(C)** OCT in the same patient. Red arrow: the origin of retinal pigment epithelium (RPE) pigment disorder; Green arrow: the initial site of RPE loss; Blue arrow: BM deletion starting site; Purple arrow: the beginning of scleral flange lengthening and thinning; Orange star: below the Zinn–Haller arterial ring; Yellow star: above the orbital cerebrospinal fluid space.

The classic categorization of PPA delineates four distinct zones, each with specific pathological and anatomical features that reflect the progression of changes in the eye associated with HM and potential complications such as glaucoma, as shown in Figure 3.

#### 4.1.1 PPA*α*

This zone is universally observed in almost all eyes with PPA, located at the very edge of PPA. It is characterized by irregular arrangement and degeneration of RPE, leading to disturbances in retinal pigment. The presence of PPAα suggests early changes in the peripapillary region, which may or may not progress to more severe forms of atrophy (7–9).

#### 4.1.2 PPA*β*

Closely associated with glaucoma pathogenesis, the PPAβ zone lacks RPE and is found adjacent to the optic disc side of PPAα (10). As one moves closer to the optic disc, atrophy of the β zone worsens, with outer layers showing RPE deletion. Progressing inward, deletions in the photoreceptor layer become apparent, and the innermost choroidal capillary layer exhibits significant thinning or absence (11). The scarcity of the choroidal capillary layer in this region results in low fluorescence on fluorescein angiography, highlighting the severity of atrophy and its potential association with glaucoma (12).

#### 4.1.3 PPA*γ*

Primarily influenced by AL, the γ zone is defined by a further loss of BM and is situated on the optic disc side of the PPAβ (13). It may encapsulate some of the larger choroidal vessels (14). The loss of BM in the γ zone signifies a more advanced stage of peripapillary atrophy, characterized by the migration of the BM opening toward the macula (15), leading to BM protrusion over the optic disc margin on the nasal side and its absence on the temporal side, adjacent to the optic disc (16).

#### 4.1.4 PPA*δ*

Exclusive to eyes with severe axial elongation, the δ zone extends from the *γ* region and is marked by a lengthened and thinned scleral flange (17). This structural alteration affects the orbital cerebrospinal fluid space and the biomechanical stability of the lamina cribrosa (sieve plate), which may contribute to the higher incidence of optic nerve damage in HM patients (18).

### 4.2 Questions about the PPA classic partition

The PPAα region is identified by disruptions in the RPE pigment, as depicted in Figure 3B, where pigment irregularities are evident between the red and green arrows. Figure 3C further illustrates uneven RPE patterns between these markers. Nevertheless, such disturbances in the RPE structure are not always conspicuous, resulting in a vague PPAα on OCT imaging.

The *β* and γ regions of PPA, as demonstrated in Figure 3, are characterized by the absence of RPE and BM, respectively. These areas extend from the green to the blue arrow and from the blue to the purple arrow, respectively. A continuous band-like appearance in OCT images is observed when the termination of the RPE or BM aligns with the nasal side of the SCA, as seen between the blue and purple arrows in Figure 3C. Conversely, a bipartite band, demarcated by the SCA, appears when these structures terminate on the temporal side, as noted between the green and blue arrows in Figure 3C. This raises the question of the appropriateness of categorizing two discontinuous segments under the same OCT section.

The definition of the *δ* region hinges on the elongation and thinning of the scleral flange, evident between the purple arrow and the optic disc margin in Figure 3. However, discerning these scleral flanges in numerous HM patients proves challenging. Out of 316 HM or PM patients studied, only 153 exhibited visible scleral flanges. Furthermore, the criteria for defining the elongation and thinning of the scleral flange remain unclear in ophthalmology. Another landmark, the Zinn–Haller arterial ring, which lies adjacent to the δ region, along with the orbital cerebrospinal fluid space, is not consistently visible on OCT images.

Consequently, we propose investigating of alternative OCT imaging markers for PPA that provide intuitive and equally significant insights into HM condition assessment.

### 4.3 Improved zoning of PPA based on OCT

Given the aforementioned observations and the weak correlation of the *α* and *β* regions with HM, we propose a new partition that omits the RPE’s marker role. This revised schema introduces three landmarks: the end of BM, the apex of SCA, and the apex of SFCA. SCA, a near-optic disc scleral curvature present in nearly all PM and the majority of HM cases, is typically near the BM’s end. SFCA, observed in the majority of the PM and a subset of HM patients, lies closer to the optic disc than SCA, typically near the junction of the dura with scleral flanges. PPA1 is a strip from the end of BM to the SCA apex on the temporal side of SCA. PPA2 extends from the end of BM to the optic disc margin on the nasal side of SCA. PPA3 is a band from the SCA apex to the optic disc edge. PPA4 spans from the SFCA apex to the optic disc rim.

#### 4.3.1 SCA

The biomolecular structure of BM is intricately composed of an inner collagen layer, an elastin layer, and an outer collagen layer. The inner and outer boundaries of the BM are enwrapped by the RPE and the choroid capillary basement membrane, respectively (19). In our meticulous analysis of numerous OCT images, we observed a consistent pattern: the scleral thickness adjacent to the SCA was notably greater than that of its temporal counterpart, as shown in Figure 1, with the degree of difference visually ascertained. This observation suggests that the formation of the SCA may follow principles similar to those underlying the development of the arch macula (20). Furthermore, prior investigations have established a correlation between the increased occurrence of arch macula and the presence of BM defects within the macula (21). This finding aligns with our own observations of BM defects peripherally to the SCA. More precisely, the BM irregularity surrounding the SCA may be more aptly described as a BM offset. Multiple studies have demonstrated that in myopic patients, the opening of the BM may shift toward the macula, and there exists a positive correlation between the degree of this deviation and the severity of myopia (22, 23). Assuming that the BM plays a crucial biomechanical role in scleral support, we hypothesize that the temporal displacement of the BM opening may lead to scleral relaxation at the site of the opening. Subsequently, the orbital fat, compressed by the enlarged eyeball, exerts a counterforce on the eyeball, causing the relaxed sclera at the opening to thicken and protrude relatively toward the eyeball. However, due to the constraining effect of the surrounding tissue of the optic disc, the BM opening on the nasal side does not exhibit significant protrusion. Consequently, in terms of gross morphology, the deformation of the eyeball is most pronounced at the SCA, resulting in a limited area of prominent deformation.

In our meticulous investigation, we observed a strong correlation between SCA and AL, as well as fundus conditions, with notable variations across all study groups. Furthermore, our multiple linear regression analysis revealed significant linear relationships between SCA and AL, BCVA, and SE. Based on our comprehensive analysis, we hypothesize that AL elongation, on the one hand, leads to further expansion of the eyeball and an increase in orbital pressure. This, in turn, exerts a compressive force on the SCA, causing it to protrude further into the eyeball. On the other hand, the biomechanical interaction between BM and the surrounding optic disc increases the traction force on the protruding sclera. These two factors, acting in concert, contribute significantly to the clinical manifestations of SCA enlargement.

#### 4.3.2 SFCA

The division of the posterior sclera occurs near the Zinn–Haller arterial ring, which provides blood flow to the ethmoid artery. This division facilitates the migration of part of the posterior sclera into the dural membrane of the optic nerve (outer portion). At the same time, the remaining segment forms the scleral flange (inner portion), extending toward the optic disc and eventually integrating into the optic nerve’s pia (24). Consequently, the Zinn–Haller arterial ring delineates the boundary between the *γ* and *δ* zones. Typically, the scleral flange is smoothly connected to the temporal sclera. Nevertheless, a notable deviation occurs in certain HM and PM cases, where the scleral flange exhibits an additional bend at this juncture, excluding the site of SCA. Predominantly, this bending is inward, though outward bending is also observed. We postulate two primary mechanisms for the emergence of SFCA: first, the biomechanical support role of the BM is compromised, leading to the scleral flange’s increased vulnerability to external pressure due to the lack of BM support; second, scleral migration to the dural membrane results in scleral thinning, heightening its susceptibility to environmental stresses. Additionally, variation in scleral pressure pre- and post-transition across this zone, shifting from a combination of IOP and intraorbital pressure to IOP and intracranial pressure, contributes to this phenomenon. Hence, SFCA is not observed in normal individuals or HM patients in the absence of these conditions. We hypothesize that in HM patients exhibiting the first two characteristics, SFCA orientation depends on the relative magnitudes of intracranial versus intraorbital pressures. Our findings indicate a predominant inward deviation of SFCA, aligning with the observation that normal intraorbital pressure (3–6 mm Hg) (25) is lower than intracranial pressure (60–200 mm H_2_O ≈ 4–15 mm Hg) (26).

Our study further demonstrates a statistically significant association between SFCA and AL, as well as fundus conditions, and effectively distinguishes between medium-to-severe PM and HM, and between severe and mild PM. When SFCA’s absolute value is considered to neutralize lateral pressures, a marked difference is observed between medium-to-severe PM and either mild PM or HM. However, multiple linear regression analysis did not establish a significant linear correlation between SFCA and AL, BCVA, and SE. This outcome may be attributed to two factors: First, elongation of the AL contributes to increased eyeball enlargement, thereby elevating orbital pressure and facilitating protrusion of the sclera in proximity to the SCA. Second, the scleral flange maintains a stable position relative to the optic disc, due to the equilibrium between internal and external pressures, as well as the constraining effects of nasal tissue at the optic disc. The final external manifestation reveals a deviation of the scleral flange from its original trajectory, resulting in a bending away from the sclera in the vicinity of the temporal side of the SCA. It is crucial to emphasize that the SFCA does not represent a literal bending of the scleral flange itself. Instead, it characterizes the second segment of the observed bending phenomenon within the sclera near the SCA.

#### 4.3.3 PPA1

All enhanced partitions of the PPA were constructed in the absence of the RPE–BM–choroid complex. Specifically, PPA1 falls within the classical *γ* zone, commencing at the ending point of BM and terminating at the vertex of the SCA.

In our study, we observed significant correlations between PPA1 and AL, as well as between PPA1 and fundus conditions. Notably, individuals in the HM group exhibited the lowest PPA1 values, whereas in the PM group, PPA1 gradually augmented as the condition advanced. Furthermore, our multiple linear regression analysis revealed a profoundly significant linear association between PPA1 and AL, BCVA, and SE. We postulate that two primary factors may underlie these observations. First, in the context of the elongated posterior bulbar wall observed in HM, the thicker sclera at the site of the SCA likely contributes to a more fixed position of the SCA relative to the overall eyeball wall. Second, AL augmentation enlarges the BM opening, resulting in a gradual shift of the BM toward the temporal side on OCT images. Consequently, we propose that PPA1 could serve as a reliable indicator, similar to the BM opening distance (27), for prognostication of HM patient outcomes.

#### 4.3.4 PPA2

In the absence of elongated and thinner scleral flanges, PPA2 may constitute a portion of or be equivalent to the entire *γ* region. Conversely, when elongated and thinner scleral flanges are present, PPA2 may extend further to encompass the *δ* region. PPA2 originates at the ending point of Bruch’s membrane and terminates at the edge of the optic disc.

In our study, we observed correlations between PPA2 and AL, as well as PPA2 and fundus conditions. Notably, PPA2 values were lower in the HM group than in the PM group. Furthermore, within the PM group, PPA2 gradually increased as the disease progressed. However, our multiple linear regression analysis revealed no statistically significant linear relationship between PPA2 and AL, and between BCVA and SE. We hypothesize that two primary factors may underlie these observations. First, in instances where BM “crosses” the SCA and PPA1 is absent, PPA2 demonstrates a strong concordance with the clinical significance of the classical partition *γ* or BM opening distance. This consistency suggests that PPA2 may serve as a potent predictor of HM severity. Second, in cases where BM terminates on the temporal side of SCA and PPA1 is present, the thicker sclera proximate to the SCA and the constraint exerted by the surrounding optic disc tissue limit scleral deformation in response to AL changes. Consequently, the length of PPA2 cannot be supplemented by the temporal migration of BM observed in the first scenario. Therefore, the correlation between PPA2 and HM severity is attenuated.

#### 4.3.5 PPA3 and PPA4

PPA3 does not align with the classical partitions of γ or *δ* regions, originating at the SCA apex and terminating at the optic disc edge. PPA4 aligns closely with the classical δ zone, characterized by an elongated and attenuated scleral flange. It begins at the SFCA vertex and concludes at the optic disc’s boundary.

Our findings indicate a distinct correlation between PPA3 and PPA4 and AL, ocular fundus conditions. Although PPA3 and PPA4 showed no difference between moderate and severe PM, a marked contrast was observed between moderate-to-severe PM and mild PM or HM (for PPA4, *p* ≤ 0.1 between severe PM and mild PM). Further, multiple linear regression analysis did not reveal a significant linear correlation between PPA3 and PPA4 and AL, BCVA, and SE. This phenomenon could be attributed to the increased thickness of the sclera near the SCA and to the scleral flange being constrained by the optic disc edge tissues. Consequently, scleral deformation due to AL alterations in this region appears to be comparatively restrained and likely only manifests during the initial and intermediate stages of HM. Deformations in the sclera during the advanced phases are more attributable to changes in the posterior pole sclera area. Furthermore, the MSFT outcomes corroborate that AL categorization does not influence MSFT metrics.

### 4.4 Clinical application potential of the modified partition of PPA

Our findings elucidate that numerous parameters within the modified partition of PPA, encompassing the SCA, SFCA, and PPA1-4, exhibited pronounced trends correlating with increased AL, diminished BCVA, and reduced SE. Notably, SCA and PPA1 demonstrated exceptionally robust correlations with AL, BCVA, and SE. These compelling statistical findings suggest that SCA and PPA1 are potential biomarkers for future diagnosis, therapeutic intervention, and prognostic evaluation of HM and PM.

These observations support the integration of quantitative PPA metrics into clinical practice to enhance diagnostic precision, enable personalized therapeutic strategies, and refine prognostic assessments. By quantifying SCA and PPA1, clinicians can obtain an objective evaluation of myopic strain and fundus pathology, thereby facilitating earlier intervention and improving risk stratification. For instance, patients exhibiting markedly elevated SCA and PPA1 values may be considered for more aggressive management, such as posterior scleral reinforcement or anti-VEGF therapy, to mitigate disease progression. Furthermore, the strong associations between these parameters and established functional and structural indices imply that SCA and PPA1 could serve as surrogate endpoints in future clinical trials, thereby enabling efficient evaluation of novel interventions. Although longitudinal validation is required, the baseline enlargement of SCA and PPA1 may indicate a faster rate of visual field loss and fundus deterioration, providing a quantitative tool for patient-specific prognosis estimation. Beyond immediate clinical utility, the refined PPA partition system offers a standardized, reproducible framework for fundamental research into the pathogenesis of HM and PM. By correlating parameter dynamics with underlying mechano-biological processes—such as scleral thinning, choroidal degeneration, and biomechanical stress—researchers can elucidate key drivers of disease progression. This methodology also opens avenues for investigating interactions between ocular structural changes and systemic factors, including intraocular pressure fluctuations and vascular perfusion, thereby generating novel hypotheses regarding myopic complications and potential therapeutic targets.

## 5 Limitation

Our investigation was conducted at a single tertiary referral center, entailing an inherent selection bias toward more severe or treatment-refractory cases of high myopia. Despite comprehensive capture of routinely available clinical parameters, we lacked data on potentially pivotal confounders—including germline genetic predisposition, cumulative near-work exposure, lifetime outdoor activity, and cardiometabolic comorbidities—that could introduce both measured and unmeasured residual confounding. As a retrospective cross-sectional study, the present findings represent associations assessed at a single time point, which precludes inferring causal relationships or temporal sequences between morphological biomarkers and disease progression. Consequently, the observed relations between quantitative morphological biomarkers and visual outcomes may overestimate or underestimate true effect sizes. Moreover, including both eyes from eligible participants, while maximizing statistical power and reflecting the bilateral nature of the disease, may introduce bias. Additionally, our cohort was overwhelmingly composed of Han Chinese individuals; this demographic homogeneity limits the generalizability of our findings to other ethnicities, in whom axial-length distribution, scleral biomechanics, and predisposition to pathological myopia may differ materially. Therefore, extrapolation of these cross-sectional associations to infer dynamic disease trajectories should be approached with caution, and the stability and predictive utility of the identified biomarkers require validation in longitudinal settings. Multicenter, prospective studies with genetically diverse populations and standardized environmental exposure metrics are warranted to validate and refine the present conclusions.

## 6 Conclusion

In summary, our study introduces a modified PPA partitioning system that enhances diagnostic, therapeutic, and prognostic capabilities in the management of HM and PM. The integration of SCA and PPA1 as key biomarkers offers a paradigm shift in the clinical approach to these conditions, with the potential to improve patient outcomes and quality of life. Future research should focus on validating the prognostic value of these parameters in diverse patient populations and exploring their applications in the development of novel therapeutic strategies.

## Data Availability

The original contributions presented in the study are included in the article/supplementary material, further inquiries can be directed to the corresponding author/s.
